# Low cut-off value of serum (1,3)-beta-d-glucan for the diagnosis of *Pneumocystis* pneumonia in non-HIV patients: a retrospective cohort study

**DOI:** 10.1186/s12879-021-06895-x

**Published:** 2021-11-29

**Authors:** Jumpei Taniguchi, Kei Nakashima, Hiroki Matsui, Tomohisa Watari, Ayumu Otsuki, Hiroyuki Ito, Yoshihito Otsuka

**Affiliations:** 1 Department of Pulmonology, Kameda Medical Center, Kamogawa, Chiba Japan; 2 Department of Clinical Epidemiology and Health Economics, School of Public Health, The University of Tokyo, Tokyo, Japan; 3 Clinical Research Support Office, Kameda Medical Center, Kamogawa, Chiba Japan; 4 Department of Clinical Laboratory, Kameda Medical Center, Kamogawa, Chiba Japan

**Keywords:** Cut-off value, Diagnostic performance evaluation, Non-HIV *Pneumocystis* pneumonia, Retrospective cohort study, Serum (1,3)-beta-d-glucan

## Abstract

**Background:**

Non-human immunodeficiency virus (HIV) *Pneumocystis* pneumonia (PCP) is a fulminant disease with an increasing incidence. The serum beta-d-glucan (BDG) assay is used as an adjunct to the diagnosis of PCP; however, the cut-off value for this assay is not well-defined, especially in the non-HIV PCP population. Therefore, we aimed to identify the assay cut-off value for this population.

**Methods:**

In this retrospective observational study, we reviewed the medical records of all patients (≥ 18 years old) with clinical suspicion of PCP who underwent evaluation of respiratory tract specimens between December 2008 and June 2014 at Kameda Medical Center. We created a receiver operating characteristic curve and calculated the area under the curve to determine the cut-off value for evaluating the inspection accuracy of the BDG assay.

**Results:**

A total of 173 patients were included in the study. Fifty patients showed positive results in specimen staining, loop-mediated isothermal amplification assay, and polymerase chain reaction test, while 123 patients showed negative results. The receiver operating characteristic analyses suggested that the BDG cut-off level was 8.5 pg/mL, with a sensitivity and specificity of 76% and 76%, respectively.

**Conclusions:**

The Wako-BDG cut-off value for the diagnosis of non-HIV PCP is 8.5 pg/mL, which is lower than the classical cut-off value from previous studies. Clinicians should potentially consider this lower BDG cut-off value in the diagnosis and management of patients with non-HIV PCP.

*Trial registration*: The participants were retrospectively registered.

**Supplementary Information:**

The online version contains supplementary material available at 10.1186/s12879-021-06895-x.

## Background

Pneumocystis pneumonia (PCP) is a fulminant infectious disease that occurs in immunocompromised individuals [1]. Human immunodeficiency virus (HIV)-infected patients are at the highest risk of PCP; however, non-HIV-infected immunocompromised patients, including those with hematologic malignancies, those receiving immunosuppressive medications for the treatment of collagen disease, and those with solid cancers, have a substantial risk of developing PCP [2]. In addition, non-HIV PCP shows a high mortality rate (30–60%), which has increased in recent years [1, 3].

Microscopic identification of *Pneumocystis jirovecii* with staining of specimens has been the gold standard for several decades [4]. Recently, because of their higher sensitivity, *Pneumocystis*-specific polymerase chain reaction (PCR) assays have also been developed for the detection of *Pneumocystis* in induced sputum or bronchoalveolar lavage fluid specimens [5]. However, since high-quality respiratory samples cannot be obtained in up to half of all patients because of severe conditions such as hypoxemia [6], the serum beta-d-glucan (BDG) assay can be used as an adjunct to the diagnosis of PCP [7]. This test generally has good sensitivity in HIV-infected patients with PCP [8], and shows a high negative predictive value, making it unlikely that a patient with a negative serum BDG result has PCP [9]. However, the cut-off values for this test are not well-defined, and the sensitivity of the assay may be lower in non-HIV-infected patient populations with a smaller *Pneumocystis* burden [10, 11]. A previous study reported a cut-off value of 31.1 pg/mL with the Fujifilm Wako assay, and this report included both HIV-infected and non-HIV-infected patients [12]. The serum BDG cut-off value in non-HIV PCP is thought to be lower than that in HIV-PCP, and several studies have been conducted to clarify the diagnostic value of serum BDG measurements in non-HIV PCP [13, 14]. However, the cut-off value for the diagnosis of non-HIV PCP is still controversial owing to the differences in test accuracies and patient backgrounds.

Thus, in this study, we assessed the clinical and analytical performance of the serum BDG assay in the diagnosis of PCP in non-HIV-infected patients and aimed to identify the cut-off value for this population.

## Methods

### Study population

We retrospectively enrolled consecutive patients (≥ 18 years old) who underwent both serum BDG assay and the evaluation of respiratory tract specimens for the diagnosis of PCP between December 2008 and June 2014 at the 917-bed Kameda Medical Center in Japan. The inclusion criteria were as follows: (1) patients (≥ 18 years old) who were suspected to have PCP based on immunocompromised status, clinical symptoms such as dyspnea, cough, and fever, and radiological findings compatible with PCP (bilateral diffuse interstitial infiltrates on chest radiographs or bilateral ground glass opacities on chest computed tomography), and (2) underwent the nucleic acid amplification test (a loop-mediated isothermal amplification (LAMP) assay and/or qualitative PCR) for the detection of *Pneumocystis jirovecii* in respiratory specimens (bronchoalveolar lavage fluid and/or induced sputum). Exclusion criteria were as follows (1) positive results in the HIV antibody test, (2) did not undergo serum BDG assay.

The serum BDG assay results, which were collected within three days before the respiratory tract specimens were tested, were used for the analysis. In addition to LAMP and/or qualitative PCR, microscopic stainings (Grocott methenamine silver stain and/or Diff-Quick™) were performed when available at the discretion of the attending physician. PCP was diagnosed based on the presence of all the following criteria: (1) positive conventional staining (Grocott methenamine silver or Diff-Quick™) or nucleic acid amplification test (LAMP or PCR) of respiratory specimens; (2) chest radiography or computed tomography (CT) findings compatible with PCP, such as bilateral ground-glass opacity; and (3) compatible clinical symptoms, including dyspnea, cough, and fever.

The following patient demographics and clinical variables were collected: age, sex, underlying diseases, and serum BDG value. This retrospective cohort study was reviewed and approved by the Research Ethics Committee of Kameda Medical Center (#20-147). The informed consent was waived by the Research Ethics Committee of Kameda Medical Center because retrospective data was collected from hospital records.

### BDG assay

The WAKO β-d-Glucan test (Fujifilm Wako Pure Chemical Corporation, Tokyo, Japan), a kinetic turbidimetric assay, was used to measure BDG levels. Briefly, 100 µL of serum was added to 900 µL of the pre-treatment solution. After incubation for 10 min at 70 °C, the sample was cooled on ice. The *Limulus* amoebocyte lysate reagent was then added to 200 µL of the pre-treated sample. Kinetic turbidimetry results were measured after 90 min at 37 °C using an MT-6500 toxinometer with a single extension module (Fujifilm Wako Pure Chemical Corporation, Tokyo, Japan). The BDG concentration was calculated by comparing the gelation time to that on a calibration curve provided by the manufacturer for each lot.

### LAMP assay

The LAMP assay was conducted with the primers F3, B3, FIP, BIP, FL, and BL, as previously described [15, 16]. The LAMP reaction was performed with a Loopamp DNA amplification kit® (Eiken Chemical, Tokyo, Japan) using reaction mixtures composed of 40 pmol each of primers FIP and BIP, 5 pmol each of primers F3 and B3, 20 pmol each of primers FL and BL; 12.5 μL of 2 × reaction mixture; 1 μL of Bst deoxyribonucleic acid (DNA) polymerase; 2 μL of DNA sample; and distilled water up to a final volume of 25 μL. The mixtures were incubated at 61 °C for 60 min (Realoop-30; Eiken Chemical) and then heated at 80 °C for 2 min to terminate the reaction. The positive control solution for *Pneumocystis jirovecii* plasmid DNA (6 × 10^6^ copies per tube) [15] and negative control solutions of Tris–EDTA buffer were also prepared with each assay. LAMP products were detected by real-time turbidity detection using a Loopamp EXIA® (Eiken Chemical, Tokyo, Japan).

### PCR assay

Qualitative PCR was performed using previously described primers [17] and a previously reported protocol [18].

### Statistical analyses

For the descriptive statistics of patient characteristics, categorical variables are shown as numbers (percentages) and continuous variables as medians (25th–75th percentile). The differences in background characteristics between PCP and non-PCP patients were compared using the t-test for continuous variables and the chi-square test for categorical variables. Statistical significance was defined as p < 0.05.

To evaluate the inspection accuracy of the BDG assay, a receiver operating characteristic curve was created, and the area under the curve was calculated. The Youden Index [19] was then used to calculate the test cut-off point that best balanced the test sensitivity and specificity. During data processing, test values below the detection limit for β-d-glucan (< 5 pg/mL) were regarded as 0.0 pg/mL. We compared the sensitivity, specificity, positive predictive value, and negative predictive value (NPV) based on the cut-off point of this study and a previous study including patients with HIV. We obtained 95% confidence intervals of sensitivity, specificity, positive predictive value, NPV, area under the curve, and BDG cut-off point using the 2000 bootstrap procedure. Statistical analyses were performed using R software (version 3.2.3, R Development Core Team, https://www.r-project.org/) and the pROC [20] and epiR (CRAN-Package epiR (r-project.org)) packages.

## Results

**Fig. 1:**
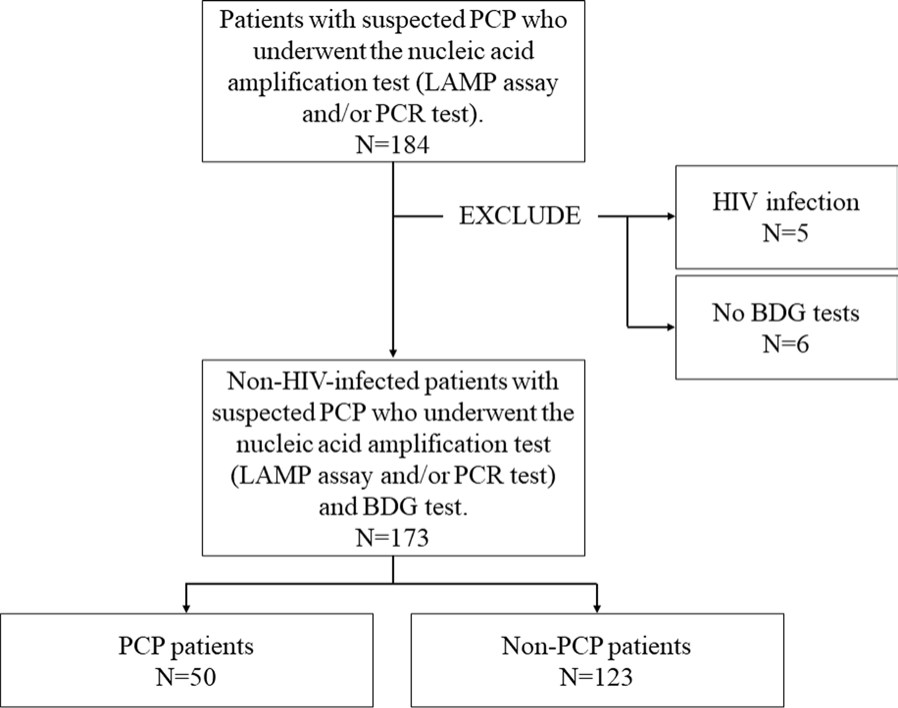
Patient selection flowchart. *BDG* beta-d-glucan, *HIV* human immunodeficiency virus, *LAMP* loop-mediated isothermal amplification, *PCR* polymerase chain reaction, *PCP* *Pneumocystis* pneumonia

Figure 1 shows the patient selection flowchart. A total of 184 patients were enrolled in this study and underwent the nucleic acid amplification test (LAMP assay and/or the PCR test). Five patients were excluded because of HIV infection. Six patients were excluded because they did not undergo a serum BDG test. Thus, a total of 173 patients were included in this study. Fifty patients showed positive results in specimen staining, LAMP assay, and PCR tests (specimen staining, 17 cases; PCR test, 27 cases; and LAMP assay, 28 cases), while 123 patients showed negative results (non-PCP) (Fig. 1). Among patients with PCP, 2 patients (2/50; 4%) received prophylactic administration of trimethoprim-sulfamethoxazole.

The demographic and clinical characteristics of the patients included in this study are summarized in Table 1. The median age of patients with positive PCP test results was 71.5 (interquartile range: 65.2–78.0) years and that of patients with negative PCP tests results was 71.0 (interquartile range: 59.0–79.0) years. Autoimmune disorders were the most frequent underlying diseases in both groups, with 52.0% of the patients in the PCP group and 39.8% of those in the non-PCP group showing autoimmune diseases. The second most common underlying disease was hematological malignancy (18.0% and 19.5%, respectively), followed by solid tumors, idiopathic interstitial pneumonia, and others (diabetes mellitus, chronic heart failure, liver failure, etc.). The means for age, sex, and underlying diseases did not show any significant differences between the PCP and non-PCP groups. The median serum BDG level was higher in the PCP group than in the non-PCP group (19.5 pg/mL vs. 0.0 pg/mL, p < 0.001). Box plots of the BDG levels in the PCP group and the non-PCP group are shown in Additional file 1: Fig. S1.

**Table 1:** Patient characteristics

	PCP (n = 50)	Non-PCP (n = 123)	p Value
Age, years (median ± IQR)	71.5 (65.2–78.0)	71.0 (59.0–79.0)	0.106
Females, n (%)	20 (40.0)	45 (36.6)	0.805
Underlying disease, n (%)			0.076
Hematological malignancy, n (%)	9 (18.0)	24 (19.5)	
Solid tumor, n (%)	9 (18.0)	12 (9.8)	
Autoimmune disorder, n (%)	26 (52.0)	49 (39.8)	
Idiopathic interstitial pneumonia, n (%)	3 (6.0)	24 (19.5)	
Others, n (%)	3 (6.0)	14 (11.4)	
Beta-d-glucan, pg/mL (median ± IQR)	19.5 (9.3–68.5)	0 (0.0–8.0)	< 0.001

*IQR* interquartile range, *PCP* *Pneumocystis* pneumonia

**Fig. 2:**
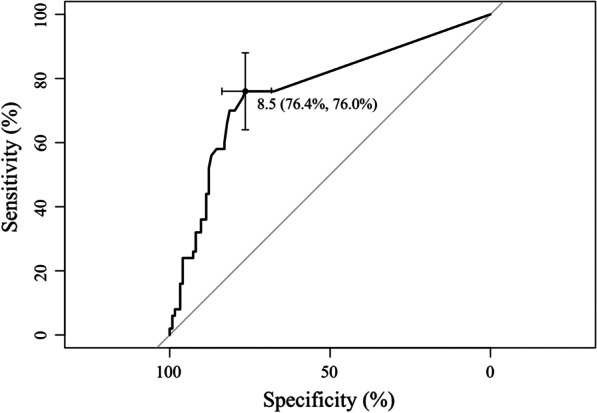
ROC curve for BDG. The area under the curve value is 0.7631. The cut-off value for BDG is 8.5 pg/mL. With this cut-off point, the sensitivity and specificity are 76% and 76% respectively. *BDG* beta-d-glucan, *ROC* receiver operating characteristic

The receiver operating characteristic curves constructed for BDG are shown in Fig. 2. The area under the curve value was 0.7631 (95% confidence interval: 0.6841–0.8421), and the cut-off value of BDG was estimated to be 8.5 pg/mL (95% confidence interval: 8.5–16.5 pg/mL). With this cut-off point, the sensitivity, specificity, positive predictive value, and NPV were 76%, 76%, 57%, and 89%, respectively (Table 2). At the classical cut-off point (31.1 pg/mL) obtained in a previous study that included a population of patients with HIV [12], the sensitivity, specificity, positive predictive value, and NPV were 36%, 89%, 58%, and 77%, respectively (Table 2). The sensitivity of the BDG cut-off point of 8.5 pg/mL was higher than that of the BDG cut-off point of 31.1 pg/mL. In addition, we performed a subgroup analysis according to the respective underlying disease groups classified in this study (Additional file 1: Fig. S2). The cut-off values in all subgroups were lower (8.0 to 16.5 pg/mL) than 31.1 pg/mL obtained in a previous study [12]. These results were consistent with those observed for all study patients (8.5 pg/mL).

**Table 2:** BDG test performance

	BDG cut-off 8.5 pg/mL % (95% CI)	BDG cut-off 31.1 pg/mL % (95% CI)
Sensitivity	76 (62–87)	36 (23–51)
Specificity	76 (68–84)	89 (83–94)
Positive predictive value	57 (44–69)	58 (39–75)
Negative predictive value	89 (81–94)	77 (70–84)

*BDG* beta-d-glucan, *CI* confidence interval

## Discussion

In this study, we evaluated the diagnostic performance of the serum Fujifilm Wako BDG assay for non-HIV-infected PCP patients. Our study findings, which were derived from 173 patients with lower airway disease who underwent evaluations for PCP, suggested a serum Wako BDG cut-off value of 8.5 pg/mL in non-HIV-infected PCP patients. In comparison with the previously reported cut-off value (31.1 pg/mL) obtained using the Fujifilm Wako-BDG assay in both HIV-PCP and non-HIV PCP populations diagnosed by microscopic staining in bronchoalveolar lavage fluid [12], the cut-off value in our study was lower. Since non-HIV PCP is a very fulminant and fatal disease [21], it is important to use a lower cut-off value with high sensitivity to prevent overlooking non-HIV PCP.

The results obtained in this study can be attributed to the following reasons. First, we excluded HIV-PCP patients to evaluate the serum Wako-BDG cut-off value for the diagnosis of non-HIV PCP. The serum BDG level in HIV-PCP is higher than that in non-HIV PCP because of the higher fungal burden in HIV patients, which correlates with serum BDG levels [22, 23]. This could be the primary reason why our cut-off value was lower than the previously reported value. Although we used Fujifilm Wako BDG in our study, the results obtained here are consistent with those of another study using another BDG assay for the diagnosis of non-HIV PCP [24]. Recently, a study comparing the diagnostic performance of the Fujifilm Wako assay and the Fungitell assay for the diagnosis of PCP was also conducted [25]. Although the study included patients with HIV, the optimal cut-off point using the Youden method was 6.222, which was similar to the results of our study. The proportion of HIV cases in the previous study was low (21/230: 9.1%), and we believe that the fact that the cut-off point indicated by the previous study is close to the results of our study indicates the robustness and generalizability of our findings. Second, we used nucleic acid amplification tests (LAMP or PCR) to diagnose PCP for all study patients. PCR and LAMP assays have been shown to have greater sensitivity than microscopic staining [5, 16]. Therefore, we could diagnose PCP more accurately and with higher sensitivity even when the fungal burden of non-HIV PCP was lower. The previous study proposing the serum Wako-BDG cut-off value of 31.1 pg/mL [12] used only microscopic staining (Grocott-Gomori methenamine stain and Calcofluor white stain) in a specimen sample and reported that PCR could detect asymptomatic colonization of *Pneumocystis jirovecii*. However, in recent clinical practice, clinicians have started using nucleic acid amplification test such as PCR for the detection of *Pneumocystis* because the sensitivity of microscopy with staining is low due to the decreased organism burden in non-HIV PCP [26]. Thus, our study evaluated the cut-off BDG value for the diagnosis of non-HIV PCP on the basis of diagnostic methods, including PCR and LAMP assays.

Our findings have important clinical implications for physicians who treat non-HIV-infected patients with PCP. The incidence and prevalence of non-HIV PCP have been increasing worldwide and considering the high in-hospital mortality rate of 30–60% associated with this form of PCP, prompt diagnosis of this condition is a critical issue that needs to be resolved [3]. Clinicians should be aware of the potentially low BDG cut-off value for the diagnosis and management of patients with non-HIV PCP. The diagnosis of non-HIV PCP may be missed or delayed with the classical cut-off value (31.1 mg/dL) due to its low sensitivity and NPV. The actual prevalence of PCP in the population composed of patients who are clinically suspected to have PCP is high (50/173 cases: 29% in this study population). There is a concern that the negative predictive value may decrease (increase in false negatives). High NPV of 89% and a low false negative of 11% with a low BDG cutoff value (8.5 mg/dL) can reduce the incidence of missed cases of PCP. Clinical practice based on a low BDG cut-off value can ensure early diagnosis of non-HIV PCP and improve the prognosis because early diagnosis and treatment are reportedly crucial for the survival of non-HIV-infected patients with PCP [27]. The Fujifilm Wako assay used in this study measures BDG concentrations using *Limulus* amoebocyte lysate, similar to the Fungitell assay, which is the only FDA (Food and Drug Admistration)-cleared and CE (Conformité Européenne)-marked assay worldwide. However, this assay has some advantages over the Fungitell assay, such as not being influenced by hemolytic, lipemic, or icteric samples [25]; therefore, it is expected to be used more frequently in the future.

This study had several limitations. First, the results of BDG tests could change clinicians’ decisions regarding the use of further tests to diagnose PCP. The relationship between the serum BDG level and PCP is already known [12]; therefore, clinicians may choose to employ additional tests for diagnosing PCP if the serum BDG level is high. This process contributes to a higher cut-off value, since it reduces the number of missed diagnoses in people with high BDG levels. Thus, the true BDG cut-off level may have been lower than the result obtained in this study. Second, PCR and LAMP assays may detect asymptomatic colonization of *Pneumocystis jirovecii*. Therefore, a positive result does not necessarily indicate PCP. There have been some studies to distinguish colonization from infection using quantitative PCR, however, it is difficult to clearly distinguish between colonization and true PCP [25, 28]. PCR and LAMP assays are thought to have high positive predictive values in patients highly suspected to have PCP [16, 29, 30]. Third, in this study, we were unable to use a direct fluorescent antibody test, which is known to be more sensitive than GMS or Diffquick stains, due to lack of access to the test [31]. However, we believe that the effect was quite small in our study, as we performed nucleic acid amplification tests (LAMP or PCR) for all patients. Fourth, in our study, there were two PCP positive cases, despite the prophylactic administration of trimethoprim-sulfamethoxazole (2/50: 4%). It is possible that prophylactic administration may have affected the BDG levels in PCP positive cases, although there is no study evaluating whether trimethoprim-sulfamethoxazole affects the cut-off point for BDG to diagnose non-HIV PCP. However, we believe that the effect was small in our study, given the small population of such patients (2/50: 4%). Finally, this was a retrospective, single-center study. Since this study did not follow a standardized protocol, we could not exclude other factors that may have contributed to the BDG cut-off value (other fungal infections, antibiotic use, concomitant bacterial infections, or dialysis) [30]. Although we provided a useful cut-off value for the Wako-BDG assay in non-HIV PCP patients, these factors might preclude the extrapolation of our conclusions to other centers. Thus, multicenter prospective studies using a standardized protocol would be ideal and are needed to confirm our results.

## Conclusions

The cut-off value of the Wako-BDG assay for the diagnosis of non-HIV PCP was 8.5 pg/mL in our study. This value was lower than the classical cut-off value of 31.1 pg/mL in a previous study including both HIV and non-HIV PCP patients. We believe that clinicians should be aware of the low BDG cut-off value for potential use in the diagnosis and management of patients with non-HIV PCP. Further multicenter prospective studies with standardized protocols are needed to confirm our results.

## Supplementary Information

**Additional file 1: Fig. S1.** Box plots of the beta-D-glucan levels in the PCP group (left) and the non-PCP group (right). **Fig. S2.** ROC curve for BDG by background disease. The cut-off value for BDG is 10.5 pg/mL with sensitivity and specificity of 89% and 92%, respectively in hematological malignancy (A) 8.0 pg/mL with sensitivity and specificity of 78% and 83%, respectively, in solid tumor (B) 16.5 pg/mL with sensitivity and specificity of 65% and 82%, respectively, in autoimmune disorder (C) 13.5 pg/mL with sensitivity and specificity of 67% and 80%, respectively, in idiopathic interstitial pneumonia (D).

## Data Availability

The data that support the findings of this study are available from the corresponding author on reasonable request. The data are not publicly available due to privacy and ethical restrictions.

## Abbreviations

BDG: Beta-d-glucan
CI: Confidence interval
HIV: Human immunodeficiency virus
IQR: Interquartile range
LAMP: Loop-mediated isothermal amplification
NPV: Negative predictive value
PCP: *Pneumocystis* Pneumonia
PCR: Polymerase chain reaction
