# Supplementary material for: Low cut-off value of serum (1,3)-beta-d-glucan for the diagnosis of Pneumocystis pneumonia in non-HIV patients: a retrospective cohort study
Source: BMC Infect Dis. 2021 Nov 29;21:1200. doi: 10.1186/s12879-021-06895-x (PMC8628137; doi:10.1186/s12879-021-06895-x)
Supplement: Supplementary file 1 — Additional file 1: Fig. S1. Box plots of the beta-D-glucan levels in the PCP group (left) and the non-PCP group (right). Fig. S2. ROC curve for BDG by background disease. The cut-off value for BDG is 10.5 pg/mL with sensitivity and specificity of 89% and 92%, respectively in hematological malignancy (A) 8.0 pg/mL with sensitivity and specificity of 78% and 83%, respectively, in solid tumor (B) 16.5 pg/mL with sensitivity and specificity of 65% and 82%, respectively, in autoimmune disorder (C) 13.5 pg/mL with sensitivity and specificity of 67% and 80%, respectively, in idiopathic interstitial pneumonia (D). [file 12879_2021_6895_MOESM1_ESM.docx]

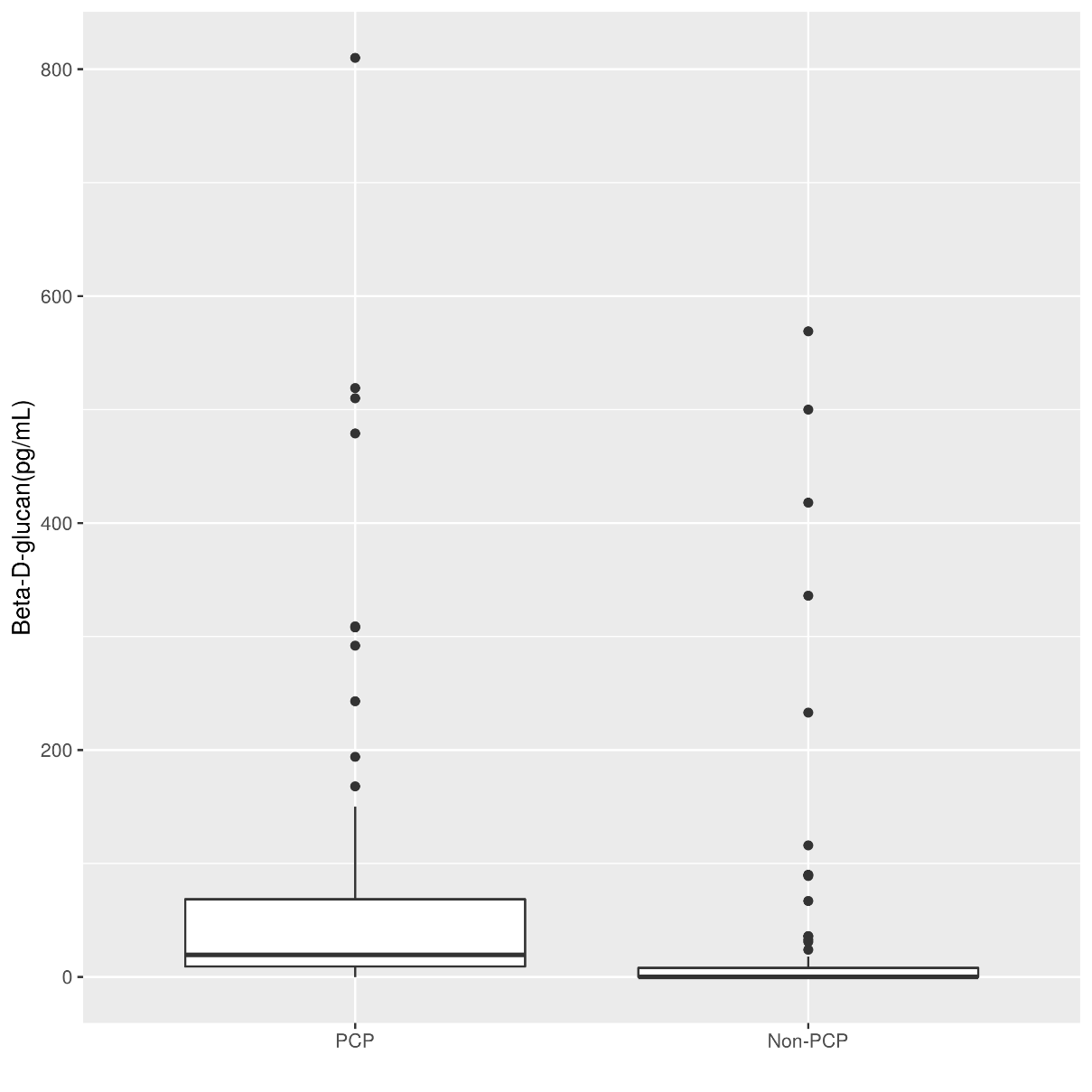


Fig. S1. Box plots of the beta-D-glucan levels in the PCP group (left) and the non-PCP group (right)

PCP: Pneumocystis pneumonia


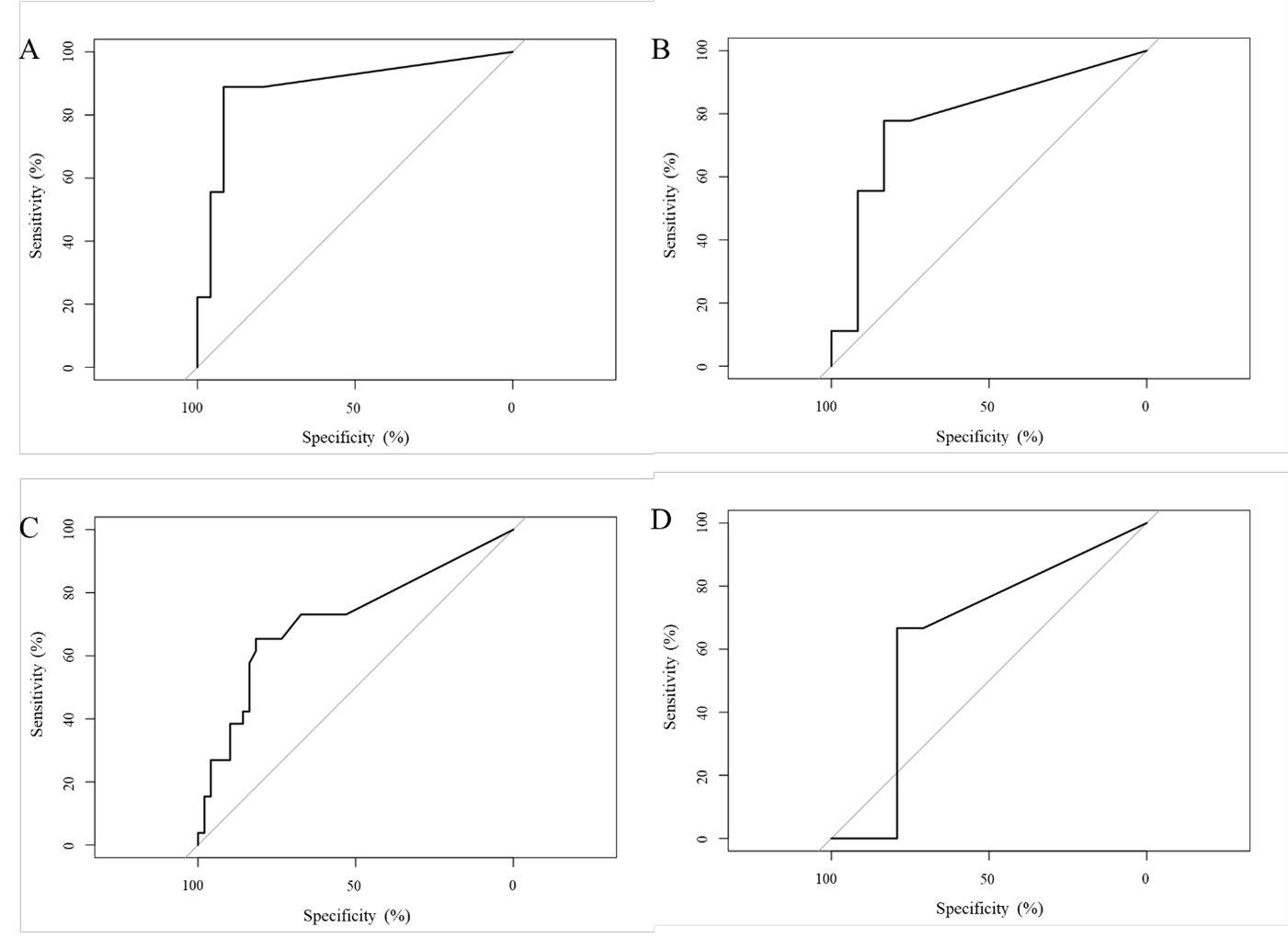


Fig. S2. ROC curve for BDG by background disease. The cut-off value for BDG is 10.5 pg/mL with sensitivity and specificity of 89% and 92%, respectively in hematological malignancy (A) 8.0 pg/mL with sensitivity and specificity of 78% and 83%, respectively, in solid tumor (B) 16.5 pg/mL with sensitivity and specificity of 65% and 82%, respectively, in autoimmune disorder (C) 13.5 pg/mL with sensitivity and specificity of 67% and 80%, respectively, in idiopathic interstitial pneumonia (D).

BDG: beta-d-glucan; ROC: receiver operating characteristic.
